# Cure Kinetics and Thermal Decomposition Behavior of Novel Phenylacetylene-Capped Polyimide Resins

**DOI:** 10.3390/polym16081149

**Published:** 2024-04-19

**Authors:** Xuhai Xiong, Hongyu Guan, Baiyu Li, Shuai Yang, Wenqiang Li, Rong Ren, Jing Wang, Ping Chen

**Affiliations:** 1 Liaoning Key Laboratory of Advanced Polymer Matrix Composites, Shenyang Aerospace University, Shenyang 110136, China; guanhongyu@stu.sau.edu.cn (H.G.); lby529i@126.com (B.L.); yangshuai2@stu.sau.edu.cn (S.Y.); lwq1269504791@126.com (W.L.); renrongsy@126.com (R.R.); jingwang1217@126.com (J.W.); 2 State Key Laboratory of Fine Chemicals, School of Chemical Engineering, Dalian University of Technology, Dalian 116024, China; chenping_898@126.com

**Keywords:** polyimide, cure mechanism, cure kinetics, thermal decomposition behavior

## Abstract

Based on a novel phenylacetylene capped polyimide (PI) with unique high-temperature resistance, its curing kinetics and thermal decomposition behavior were investigated. The curing mechanism and kinetics were studied by differential scanning calorimetry (DSC), and the activation energy (*E_a_*) and pre-exponential factor (*A*) of the curing reaction were calculated based on the Kissinger equation, Ozawa equation, and Crane equation. According to the curve of conversion rate changing with temperature, the relationship between the dynamic reaction *E_a_* and conversion rate (α) was calculated by the Friedman equation, Starink equation, and Ozawa–Flynn–Wall (O-F-W) equation, and the reaction *E_a_* in different stages was compared with the results of molecular dynamics. Thermogravimetric analysis (TGA) and a scanning electron microscope (SEM) were used to analyze the thermal decomposition behavior of PI resins before and after curing. Temperatures at 5% and 20% mass loss (*T*_5%_, *T*_20%_), peak decomposition temperature (*T_max_*), residual carbon rate (RW), and integral process decomposition temperature (*IPDT*) were used to compare the thermal stability of PI resins and cured PI resins. The results display that the cured PI has excellent thermal stability. The *E_a_* of the thermal decomposition reaction was calculated by the Coats–Redfern method, and the thermal decomposition behavior was analyzed. The thermal decomposition reaction of PI resins at different temperatures was simulated by molecular dynamics, the initial thermal decomposition reaction was studied, and the pyrolysis mechanism was analyzed more comprehensively and intuitively.

## 1. Introduction

**Figure 1:**
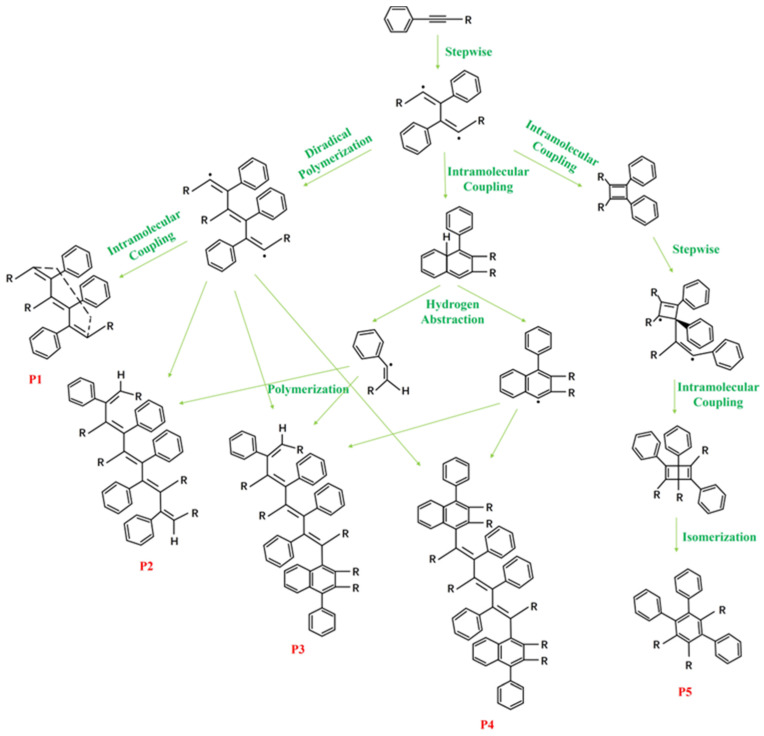
Curing mechanisms for phenylacetylene-capped PI resins (P1–P5 represent predicted cured products).

Polyimide (PI) resin is an organic material with extremely high heat resistance and can be widely used to manufacture composite structures that are resistant to temperatures as high as 300 °C [1,2,3,4,5,6], making it irreplaceable in the aerospace industry [7,8]. Among them, the heat resistance of phenylacetylene-capped polyimide resins can even reach 450 °C. They are capable of replacing aluminum alloy structures as resin-based materials in airplanes, missiles, and rockets, while reducing the weight by 15% to 30% [9]. Allowing for this praiseworthy and fascinating advantage, polyimide resins play an incomparable advantage in the aerospace field and are known as “the most promising polymer materials in the 21st century” [10,11,12]. Therefore, the curing mechanism and thermal stability of phenylacetylene-capped polyimide resin have always been the focus of research, which has attracted the extensive attention of many scholars. Chen et al. measured the curing reaction process of amide groups on the PI precursor PAA by the reaction temperature and analyzed the formation of a cured product network by FTIR [13]. Qian et al. used a compound catalyst of isopropyl peroxide and cobalt naphthalene to optimize the curing temperature of phenylethyl terminated PI, so that polyimide/carbon fiber composites could be cured at 300 °C, and the TGA characterization of the cured material and the mechanical property test of the composites showed good heat resistance and mechanical stability [14]. Cheng et al. investigated the curing behavior of the mixture of an imide monomer and acetylene monomer by combining DSC and FTIR techniques and analyzed the cured product by TGA. The results showed that the thermal stability and thermal oxidation stability of the cured product of imide monomer were greatly improved by acetylene terminating [15]. However, there is still no clear information about the microscopic curing reaction and thermal decomposition reaction mechanism of phenylacetylene-capped PI. According to the analysis of the thermal curing reaction of acetylene-terminated polyimide by Chen et al. [16], the curing reaction mechanism of phenylacetylene-capped PI was predicted as shown in Figure 1.

Undoubtedly, in addition to the molecular structure of various components in the system, the properties of polymer materials, such as *T_g_*, modulus and toughness, also depend on the curing degree and curing route, which are controlled by the curing time and curing temperature [11,17,18]. At the same time, the thermal decomposition behavior of the thermosetting polymers is also very related to the structure of the cured products. In the previous study, we characterized the isothermal curing process of PI by DMA and determined the best curing process by using the theory of the torsional weaving analysis [19], but the mechanism and kinetics of its curing reaction and the characteristics of thermal decomposition need further investigation. Therefore, the properties of polymers need to be optimized according to reliable curing and thermal decomposition kinetic parameters and the micro-mechanism during the reaction. However, the study of curing kinetics and thermal decomposition reactions is still at the stage of obtaining the best performing materials in terms of industrial engineering, and these studies do not investigate the intramolecular mechanisms from a microscopic point of view. Molecular dynamics simulations can overcome these limitations and provide a more accurate and comprehensive view of the curing mechanisms and thermal decomposition reaction processes at the atomic and molecular levels.

Therefore, this paper will analyze the curing reaction and thermal decomposition reaction process of phenylacetylene-capped PI by combining experimental tests, characterization analyses and simulation calculations. In the former study, we designed and synthesized a phenylacetylene-capped PI, investigated its curing process, and obtained the best curing process. The aim of this research is to study the curing kinetics and thermal decomposition behavior of phenylacetylene-capped PI by non-isothermal DSC, thermogravimetric analysis, and molecular dynamics simulation.

## 2. Materials and Methods

### 2.1. Experimental Section

#### 2.1.1. Experimental Materials

4-Phenylethynylphthalic anhydride (4-PEPA) and 4,4′-oxydianiline (4,4′-ODA) were obtained from Shanghai JingHui Industries, Inc. (Shanghai, China). N, N-dimethylacetamide (DMAc) solvent was obtained from Shanghai National Pharmaceutical Group, Inc. (Shanghai, China).

#### 2.1.2. Preparation of PI Resins

**Figure 2:**
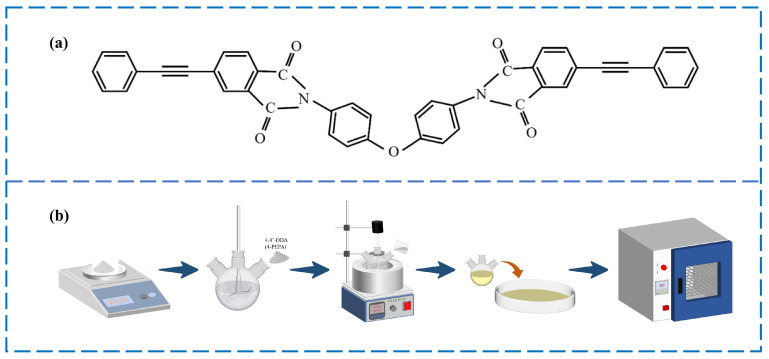
(**a**) Molecular structure of phenylacetylene-capped PI monomer; (**b**) schematic diagram of PI resin preparation process.

The PI applied in this work was made from 4,4′-oxydianiline (4,4′-ODA) with 4-phenylethynylphthalic anhydride (4-PEPA) in N, N-dimethylacetamide (DMAc) solvent. The polyimide resins were prepared by sequentially dissolving 4,4′-ODA (20.02 g, 0.1 mol) and 4-PEPA (50.45 g, 0.2 mol) into DMAc (300 mL) and mechanically stirred at ordinary temperature for about 10 h, obtaining a uniform and transparent mixture. The mixture was poured directly into tinfoil, transferred to an oven at 200 °C, and dried for 48 h to obtain a PI resin powder. Then, PI resins were cured in an oven according to the curing process of 300 °C × 2 h + 330 °C × 2 h + 350 °C × 2 h + 370 °C × 2 h. Finally, the cure resins were taken out and characterized. The molecular structure and preparation process of the PI resin are shown in Figure 2.

### 2.2. Characterization

Differential scanning calorimetry (DSC) measurements were taken with a PE Diamond instrument. About 10 mg powder was placed at the heating rate of 5~20 °C/min under a flow of N_2_ (20 mL/min).

Fourier transform infrared spectroscopy (FTIR) spectra were recorded on the IRTracer 100 infrared spectrometer of Shimadzu company (Kyoto, Japan) in Japan. The KBr pressing method was used in the testing process. The spectra were collected over the 4000~400 cm^−1^ wavenumber range at room temperature.

Thermogravimetric analysis (TGA) was conducted on PI before and after curing on the STA499F3 thermal analyzer, and the powder (about 3~10 mg) was heated from 25 °C to 800 °C at a heating rate of 20 °C/min in a N_2_ environment.

Scanning electron microscope (SEM) of SU3500 of Hitachi High-tech Corporation (Hitachinaka, Japan) was used to observe the micro-morphology of PI resins before and after curing in vacuum mode, and the voltage was 10 kV.

### 2.3. Molecular Dynamics Simulation

**Figure 3:**
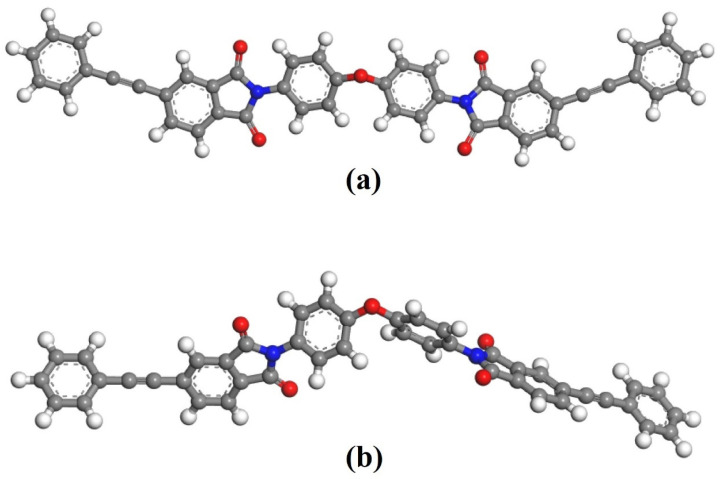
PI model establishment: (**a**) initial model; (**b**) optimized model (Among them, C atom-dark gray; H atom-light gray; O atom-red; N atom-blue, the same below).

In this work, the models of PI resins were constructed by Materials Studio (MS) 2017 software package from BIOBIA (San Diego, CA, USA) software. As shown in Figure 3a, the single-chain PI model was established. The stable structure as shown in Figure 3b was obtained through geometric optimization and energy optimization. The optimized molecular chain was twisted, and the twist angle and spatial conformation changed. Then, the curing reaction process was simulated by the Forcite tool.

In order to further study the thermal decomposition mechanism of phenylacetylene-capped polyimide molecules, the thermal decomposition process of the PI molecular chain was simulated at different temperatures by selecting the GULP module. Because the PI molecular chain needed to receive enough energy in a very short time to cause pyrolysis, the simulated temperature was obviously higher than the actual temperature, and the high temperature would not affect the study of reaction mechanism. Finally, the DMol_3_ module was selected to calculate the bond energy of the initial fracture, so as to predict the initial reaction of thermal decomposition.

## 3. Results

### 3.1. Curing Behavior and Curing Kinetics

**Figure 4:**
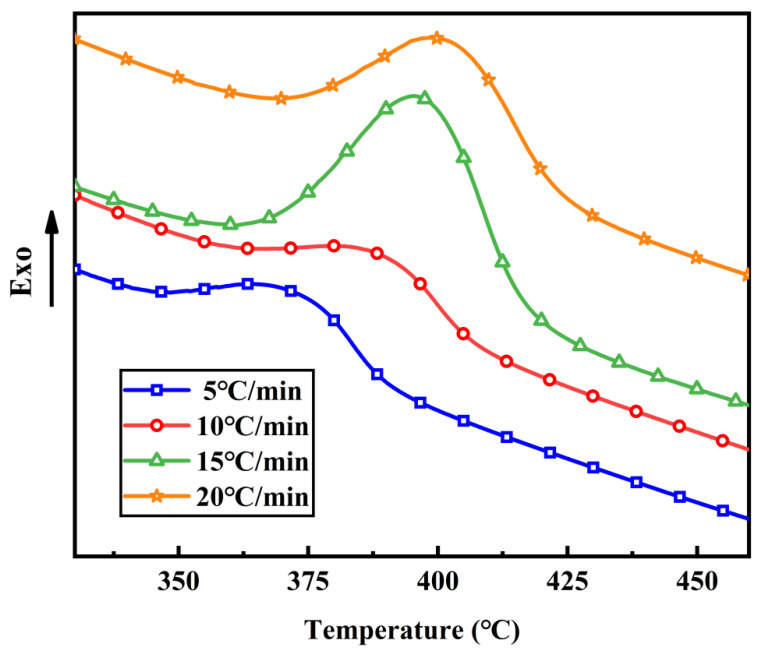
DSC curves of PI resins at different heating rates.

Figure 4 points to the dynamic DSC thermal analysis of PI resins at dissimilar heating rates (5 °C/min,10 °C/min, 15 °C/min, 20 °C/min) and the feature results are numbered in Table 1. At whole ramp rate, there is only one curing heat-releasing top appearing in the DSC curve in the wide temperature range of 340~450 °C, which indicates that only one exothermic reaction occurs. The exothermic peaks are generally rounded, which displays that the curing reaction proceeded gently. In the wake of the growth of the heating speed from 5 °C/min to 20 °C/min, the exothermic summits slowly move to the high-temperature direction, and the top shapes become sharper. The main reason is that with the addition of the heat up rate, the greater the thermal effect per unit time, the greater the temperature deviation, and the exotherm of curing reaction moves to a high temperature accordingly, which accelerates the curing reaction.

Table 1 shows the characteristic curing temperatures under dissimilar heating rates according to the dynamic DSC curves of PI resins. It can be perceived that with the increase in the heating speed, the initial curing temperature *T_i_*, the peak curing temperature *T_p_* and the finish curing temperature *T_f_* all increase.

(1) Determination of apparent activation energy and reaction order by multiple heating rate method

Apparent activation energy (*E_a_*) and order of reaction (*n*) are two highly significant curing kinetic parameters. The difficulty of the curing reaction can be judged according to the size of *E_a_*. Only if the energy of the curing system is greater than *E_a_* can the curing reaction proceed smoothly. The mechanism of the curing reaction can be analyzed according to the reaction order. The *E_a_* of the curing reaction can be calculated by the Kissinger equation [20,21,22,23] and the Ozawa equation [24,25,26], and the reaction order can be calculated by the Crane equation [27].

Kissinger equation:

(1) $$\ln\left(\frac{\beta }{T_{p}^{2}}\right)=-\frac{E_{a}}{RT_{p}}+ln(\frac{AR}{E_{a}})$$

Ozawa equation:

(2) $$\frac{d(ln\beta )}{d(1/T_{p})}=-\frac{1.052}{R}E_{a}$$

where *β* is the heating rate (°C/min), *T_p_* is the peak maximum temperature (°C), *n* is the reaction order, *R* is the gas constant, and *A* is the pre-exponential factor.

Crane equation:

(3) $$\frac{dln(\beta )}{d(1/T_{p})}=-(\frac{E_{a}}{nR}+2T_{p})$$

For the thermosetting resin, *E_a_*/*nR* >> 2*T_p_*, Equation (3) is simplified as follows:

(4) $$\frac{dln(\beta )}{d(1/T_{p})}=-\frac{E_{a}}{nR}$$

**Figure 5:**
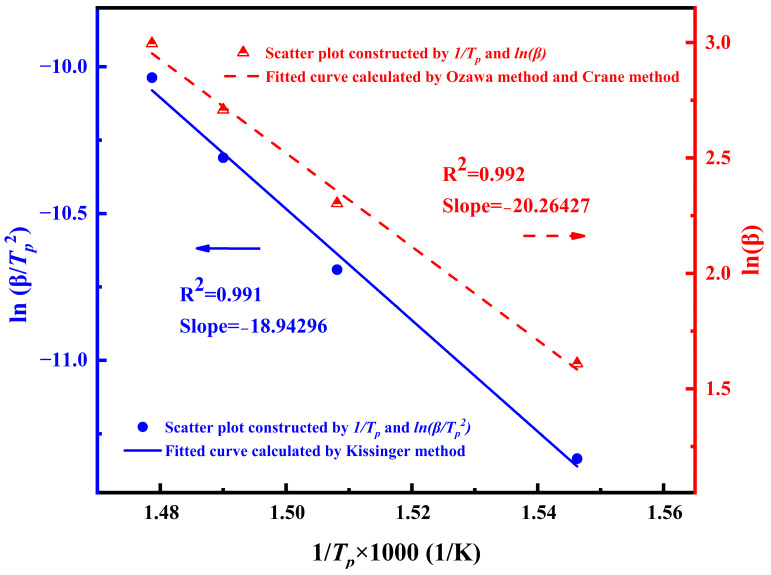
Plots of ln(*β*/*T_p_^2^*) and ln(*β*) versus 1000/*T_p_*.

According to the exothermic peak temperature (*T_p_*) of DSC at different heating rates, the typical kinetic plots of ln(*β*/*T_p_^2^*) versus 1/*T_p_* and ln(*β*) versus 1/*T_p_* were made, respectively, and the data points calculated by the two methods showed a highly linear relationship. The fitted image is shown in Figure 5.

The slope of the image obtained by linear fitting is brought into Equations (1), (2), and (4). The apparent activation energies calculated by the Kissinger equation and Ozawa equation are 157.5 kJ/mol and 160.2 kJ/mol, respectively. The reaction order calculated by the Crane equation is 0.93. It is basically a first-order reaction. This shows that the triple bond in the phenylacetylene capping agent opens during the high-temperature curing process and basically forms a polyene structure, and only a small amount of polyene structure further forms single bond [28]. In addition, the value of ln*A* can be further calculated by the Kissinger formula and *E_a_* as 20.87, and the value of the pre-exponential factor *A* is 1.16 × 10^9^.

The reaction kinetics equation of curing polyimide resin can be obtained by *n* as follows:

(5) $$\frac{d\alpha }{dt}={A(1-\alpha )}^{0.93}$$

where *α* is the degree of reaction conversion; *dα*/*dt* represents the reaction rate.

According to the equation,

(6) $$\ln\left(\frac{d\alpha }{dt}\right)=lnA-\frac{E_{a}}{RT}+nln(1-\alpha )$$

Therefore, the kinetic equation of the curing reaction is [29],

(7) $$\frac{d\alpha }{dt}=1.16\times 10^{9}\exp\left(-\frac{157,500}{RT}\right)\;{\left(1-\alpha \right)}^{0.93}$$

(2) Determination of kinetic parameters of the curing reaction by the equal conversion method

Although the Kissinger method has been applied for kinetic analysis, it only produces a single value of the activation energy for the whole process. Only when *E_a_* remained constant in the whole process could the obtained results be credible. However, in general, the reaction occurring in the course of the cure is elaborate, and the change in *E_a_* with the progress of the curing reaction is impractical. The relationship between the activation energy and conversion rate can be received by the kinetic analysis of the equal conversion rate. The analysis of this correlation helps to solve the complex mechanism of the curing process and to predict the kinetics.

**Figure 6:**
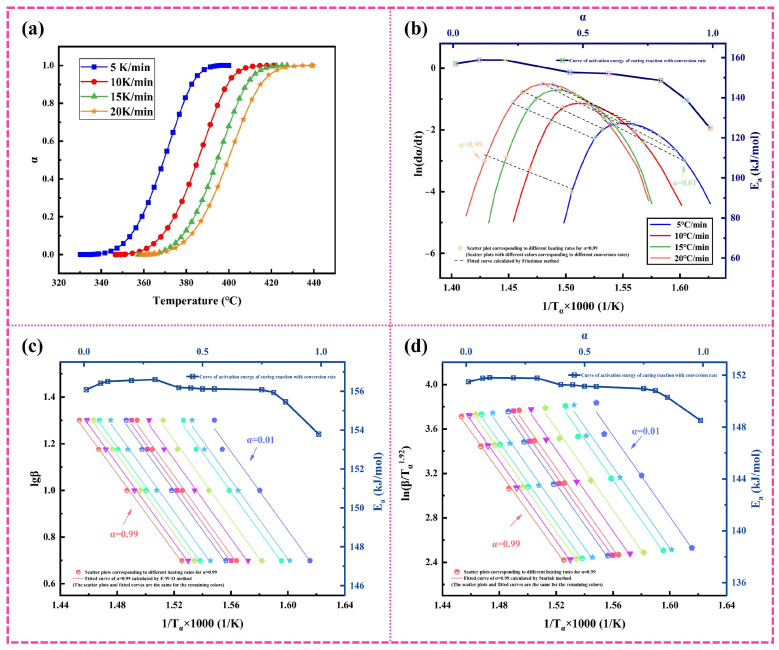
Determination of kinetic parameters of curing reaction by equal conversion methods: (**a**) fractional conversion as a function of temperature for various heating rates; (**b**) Friedman method; (**c**) F-W-O method; (**d**) Starink method.

In order to carry out the equal inversion analysis, the original DSC data of exothermic peak were converted into the curve of the fractional conversion (*α*) and temperature at different heating rates, and the corresponding figure is shown in Figure 6a. The α values were calculated by the integral of the exothermic peak according to the following equation:

(8) $$\alpha =\frac{H_{\alpha }}{H_{total}}$$

where *H_α_* is the fractional enthalpy, and *H_total_* is the total enthalpy of the cure reaction.

From Figure 6a, it can be distinctly seen that at first, all α values increase tardily with the beginning of the curing reaction, and when the resins are heated to the specified temperature, *α* values increase aggressively and then stabilize. Moreover, in order to acquire the same *α* value, the needed temperature is increased with an increasing heating rate.

Figure 6b is a dynamic model of the curing process calculated by the Friedman method [30], which is based on the following equation:

(9) $$\ln\left(\frac{d\alpha }{dt}\right)=lnA+nln\left(1-\alpha \right)-\frac{E_{a}}{RT}$$

The *E_a_* can be estimated by drawing the relationship between ln(*da*/*dt*) and 1/*T*, and the change in reaction *E_a_* with *α* can be calculated by the slope −*E_a_*/R at different conversion rates. The *E_a_* of the curing reaction presents three stages; the initial activation energy is high, the activation energy decreases slightly in the middle of the reaction, and finally, the activation energy decreases rapidly. The average activation energy calculated by this method is about 148.9 kJ/mol.

In Figure 6c,d, we adopt the Flynn–Wall–Ozawa (F-W-O) method [31] and Starink method [32] and calculate the image of 1/*T_α_* by *lgβ* and ln(*β*/*T_α_*^1.92^), respectively, to obtain the relationship between *E_a_* and *α*. The calculation formulas of the F-W-O method and Starink method are as follows:

(10) $$lg\beta =-0.4567\frac{E_{a}}{RT}-2.315+lg(\frac{AE_{a}}{G(\alpha )R})$$

(11) $$\ln\left(\frac{\beta_{i}}{T_{\alpha }^{1.92}}\right)=const-1.0008(\frac{E_{a}}{RT_{a}})$$

As displayed in Figure 6c,d, the activation energy *E_a_* changes with the degree of transformation of *α*. The images calculated by the two methods show that the fitting effect of α is good in the curing range, and the linear regression coefficients are all above 0.99. With the curing reaction, the activation energy presents three reaction stages. In the range of 0.1 ≤ *α* ≤ 0.4, the reaction is controlled by chemical methods and the activation energy is relatively high. In the range of 0.4 ≤ α ≤ 0.8, the *E_a_* value is relatively stable. The *E_a_* calculated by the F-W-O method is almost invariable around 156 kJ/mol, which is roughly in accord with the value calculated by the Kissinger equation. When the *α* exceeds 0.8, the activation energy obviously decreases, and the reaction type may change. And the *E_a_* calculated by these three methods is nearly in the range of 140~160 kJ/mol, which is very similar to Lanver’s calculation result [33].

### 3.2. Molecular Dynamics Simulation of Curing Reaction Process

**Figure 7:**
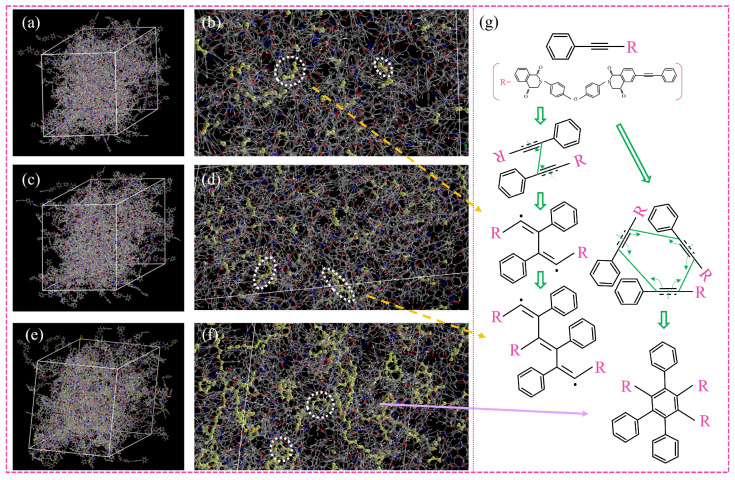
Simulation diagram of the curing reaction under different conversion rates: (**a**,**b**) the conversion rate is 0.25; (**c**,**d**) the conversion rate is 0.55; (**e**,**f**) the conversion rate is 0.95 (the reacted C≡C is shown as a stick model, and the unreacted part is shown as a line); (**g**) the pathway of phenylacetylene curing reaction (The pink letter “R” represents the omitted structure; the green arrow represents the next step of the reaction; the yellow arrow and the purple arrow indicate the molecular structures corresponding to the two cured products of the molecular dynamics simulations).

**Figure 8:**
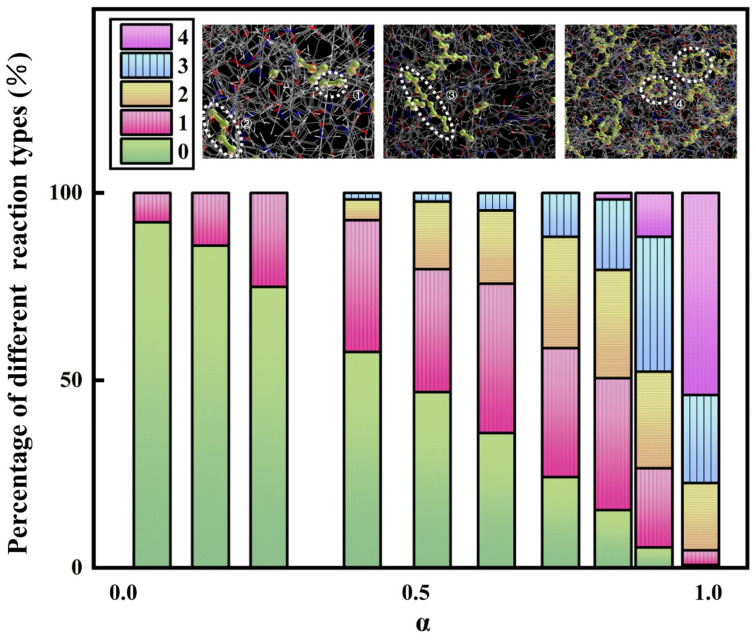
The number of different reaction types of C≡C varies with the conversion rate (Numbers 1–4 represent four bonding types of carbon-carbon triple bonds).

**Figure 9:**
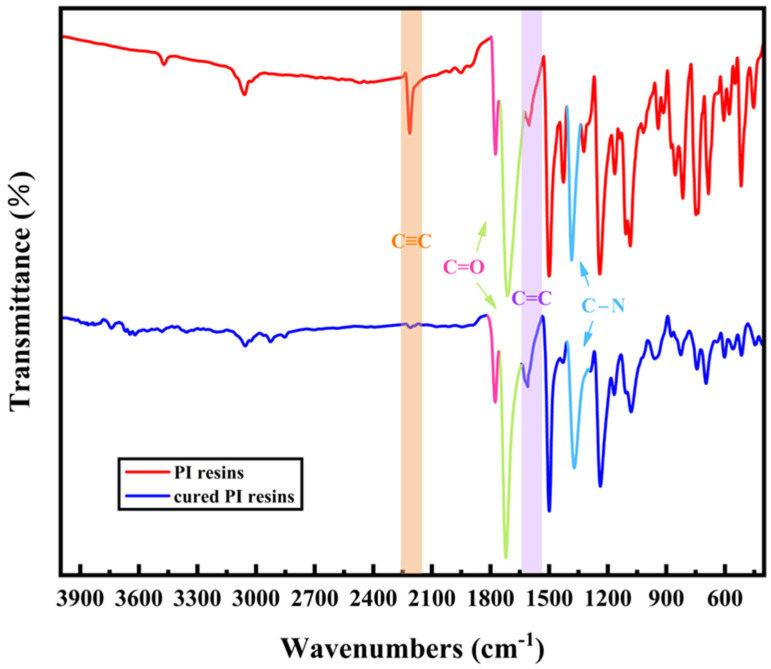
FTIR spectra of PI resins and cured PI resins (The pink-green and blue marked peaks indicate the characteristic peaks of the imide ring; Orange bars and purple bars represent changes of C≡C).

In order to verify the conjecture, the curing process of PI resins was simulated by MS software, as pictured in Figure 7. On the whole, the higher the curing degree, the more C≡C will react and the more sufficient the curing reaction will be. Specifically, the reaction is divided into three stages. First of all, C-C=C-C is generated initially. With the progress of the reaction, C≡C continues to react to form a long chain C-C=C-C=C-C. During the later stage of the reaction, the molecules gradually became longer, cyclic structures began to appear, and there were nearly no short chains left. It can be found from Figure 8, that only a small part of C≡C reacts first, and with the advancement of the reaction, the types of reactions increase, and the unreacted C≡C diminishes step by step until the reaction is over. The conversion rate corresponding to the change in reaction type is shown in Figure 9. It can be seen that when the reaction type changes, the conversion rate corresponds exactly to the three reaction stages in Figure 6b–d, which is consistent with the phenomenon shown in the simulation. At the same time, the reaction mechanism shown by the simulation is consistent with that predicted in Figure 1, and the curing mechanism is verified again.

Figure 9 shows the FTIR spectra of PI resins and cured PI resins. The curing reaction of PI resins capped by phenylacetylene is mainly that C≡C is initiated by free radicals to form -C=C-, and then C=C undergoes further complex cyclization and cross-linking reactions. Among them, the imide ring (-CO-NR-CO-) does not change during the curing reaction, so the symmetric and asymmetric C=O stretching vibration peaks at 1780 cm^−1^ and 1720 cm^−1^ and the C-N stretching vibration peak at 1380 cm^−1^ can be selected as reference peaks. The C≡C of PI resins is very obvious at 2210 cm^−1^, but the peak of the cured product is very small here and almost disappears, which shows that most C≡C undergoes crosslinking and curing reactions. The peak of C=C generated by the reaction is enhanced at 1600–1620 cm^−1^.

### 3.3. Thermal Degradation Behavior

**Figure 10:**
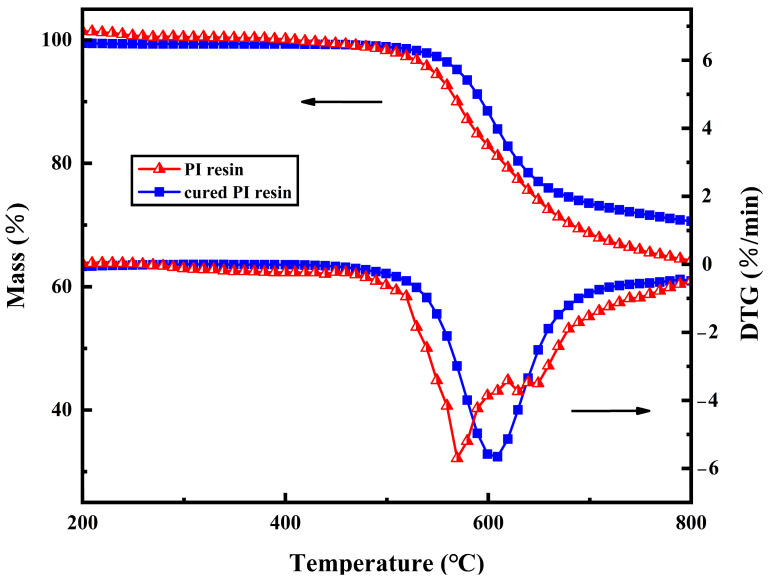
TGA curves of PI resins and cured PI resins (The arrows in the figure indicate the Y-axis corresponding to the curve).

In N_2_ atmosphere, the dynamic TG and DTG thermal analysis of PI resins before and after curing were shown in Figure 10. Temperatures with a mass loss of 5% and 20% (*T*_5%_ and *T*_20%_), decomposition peak temperature (*T_max_*), and residual carbon (RW) at 800 °C were selected as thermal stability parameters, which were abstracted in Table 2. It can be discovered that the initial decomposition temperature of PI resins and cured PI resins is above 500 °C, which shows that PI capped by phenylacetylene has excellent thermal-oxygen stability. The DTG of PI resins has two decomposition peaks; the first decomposition peak is obvious, and the second decomposition peak is smaller. This may be because the resins undergoes the decomposition reaction and curing crosslinking reaction at the same time in a high-temperature environment. Therefore, the first decomposition peak is the thermal decomposition and thermal volatilization of PI resin monomers, which have relatively low reaction temperatures. As the reaction proceeds, some resins react to form cured products, and the second exothermic peak is the decomposition peak of the cured products. Comparing *T*_5%_, *T*_20%_, and *T_max_* of the PI decomposition, it is found that the cured PI resins needs a higher temperature to reach the decomposition degree of PI before curing, that is to say, the thermal decomposition temperature of the cured PI is higher. This obviously shows that curing will increase the thermal stability of the PI resin and inhibit its decomposition. The RW of PI resins before and after curing is similar because their C content is the same. However, the RW of cured PI resins increased by 6.3% compared with that before curing, which indicated that some C atoms did not combine with other atoms to form a small molecular gas to escape, and further proved that the cross-linking structure improved the stability of the resins.

**Figure 11:**
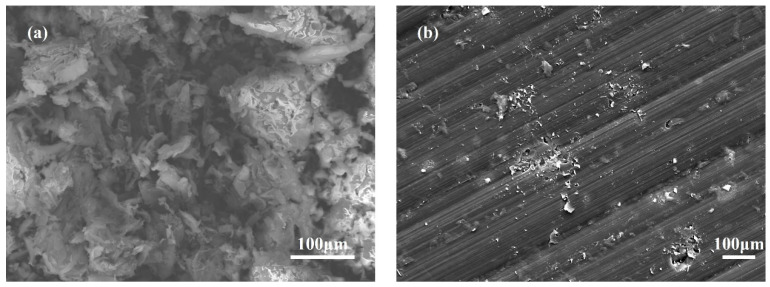
SEM of (**a**) PI resins; (**b**) cured PI resins.

As can also be found in Table 2, the residual carbon rate of cured PI is 70.6% at 800 °C, which may be related to the crosslinking density, free volume content, and heat-resistant aromatic number of the cured products. From the molecular level, when the temperature far exceeds *T_g_*, the curing network of PI will expand and the gap between the molecular chains will increase, which is beneficial to the diffusion of volatile small molecules in the initial stage of decomposition. The network structure of an extremely crosslinked PI system is overly rigid, and cracks will be formed during decomposition. This slight carbon layer is not enough to preclude mass loss and heat transfer. For the sake of testing the above hypothesis, we observed the microstructure of carbon produced by the degradation of cured resins with the SEM in Figure 11. The figure shows the external surface morphology of cured PI resins. Obviously, the PI surface presents a compact, rigid, and brittle structure.

### 3.4. Thermal Stability and the Activation Energy of Thermal Decomposition

**Figure 12:**
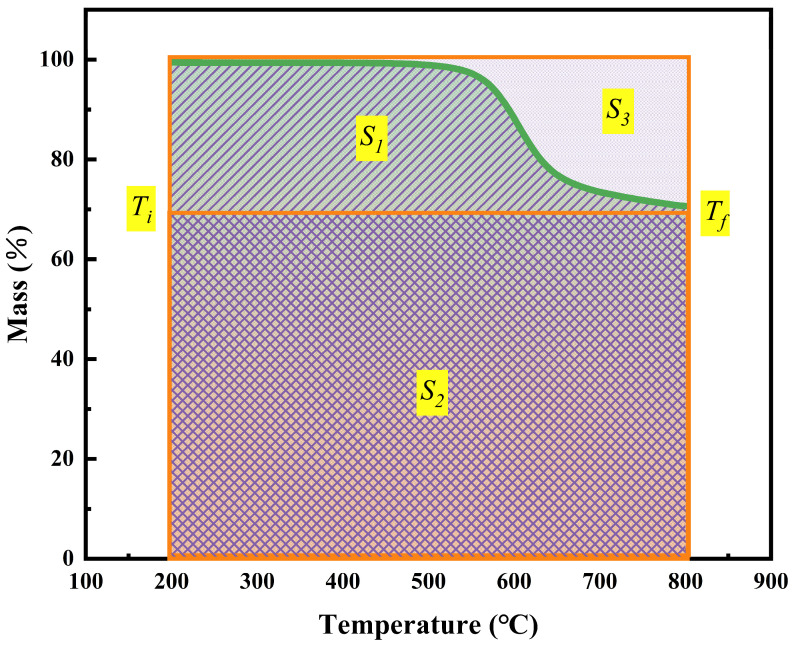
Schematic representation of *S*_1_ (single diagonal area), *S*_2_ (double diagonal grid area), and *S*_3_ (the area of orange box in the figure—the area enclosed by the curve to the X-axis in the range *T_i_* to *T_f_*) for A* and K*.

The integral procedural decomposition temperature (*IPDT*) is a universal method for evaluating the inherent thermal stability of different materials [34,35,36]. It is related to the volatile compounds of polymer materials, and the experimental results are not impacted by the experiment conditions and the particle size, shape, and appearance of the sample. The IPDT was calculated by Equation (12).

(12) $$IPDT=A^{*}K^{*}\times \left(T_{f}-T_{i}\right)+T_{i}$$

where A* and K* are the area ratios of the total experimental curve defined by the total TGA thermogram. *T_i_* is the initial experimental temperature, and *T_f_* is the final experimental temperature. During this work, the *T_i_* and *T_f_* were 200 °C and 800 °C, respectively. A* and K* can be calculated using Equations (13) and (14). The values of *S*_1_, *S*_2_, and *S*_3_ are shown in Figure 12.

(13) $$A^{*}=\frac{S_{1}+S_{2}}{S_{1}+S_{2}+S_{3}}$$

(14) $$K^{*}=\frac{S_{1}+S_{2}}{S_{1}}$$

**Figure 13:**
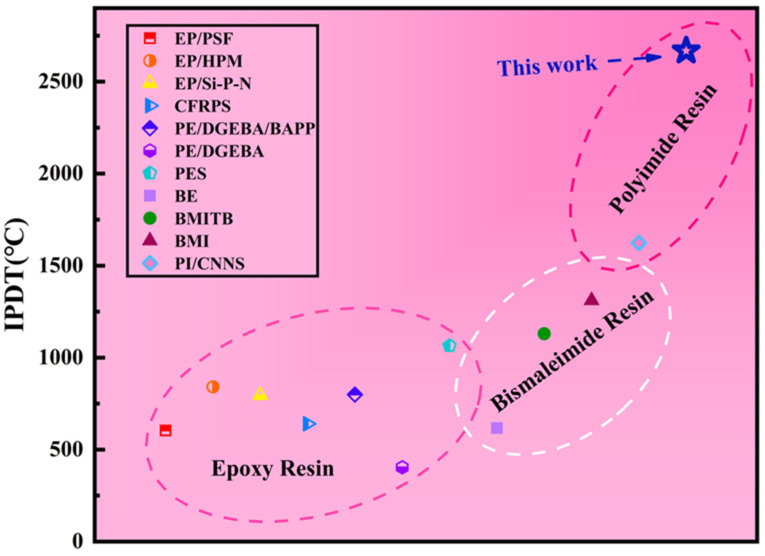
Comparison of *IPDT* with other previously reported resins [35,37,38,39,40,41,42,43,44,45,46] (Icons of different colors represent the IPDT values corresponding to different values).

Table 2 shows that the IPDT values of PI before and after curing are 2127.7 °C and 2667.9 °C, respectively. Because PI resins have more aromatic nuclei and heterocyclic rings after curing, they promote the shaping of carbon, thereby enhancing the immanent thermal stability of the polymer network. Compared with the IPDT of epoxy resin and bismaleimide resin (Figure 13), polyimide showed outstanding advantages, which further proved its excellent stability.

In order to deeply understand the thermal decomposition of the polyimide resin, Coats–Redfern model was selected to analyze the thermal decomposition process. For *n*-order reactions, under dynamic conditions,

(15) $$\frac{d\alpha }{dt}=(\frac{A}{\beta })exp(-\frac{E_{a}}{RT}){\left(1-\alpha \right)}^{n}$$

where *α* is the conversion rate of the decomposition reaction, and the residual carbon is deducted during calculation. It is considered that the conversion rate of the decomposition reaction at 800 °C is 100%, *k* is the rate constant, *n* is the reaction order, *β* is the heating rate, *E_a_* is the decomposition activation energy, and *A* is the frequency factor.

Integrating Equation (15), when *n* = 1,

(16) $$\ln\left(-\frac{\ln\left(1-\alpha \right)}{T^{2}}\right)=\ln\left(\frac{AR}{\beta E_{a}}\left(1-\frac{2RT}{E_{a}}\right)\right)-\frac{E_{a}}{RT}$$

Ignoring the 2*RT*/*E_a_* term, plot 1/*T* with ln(−ln(1 − *α*)/*T*^2^). If it is a straight line, the activation energy *E_a_* can be calculated from the slope of the straight line, and then the frequency factor *A* can be calculated. Because the thermal decomposition of the cross-linked network belongs to random chain-breaking decomposition, there will be no obvious mistakes when the thermal decomposition kinetics is assumed to be a first-order reaction.

**Figure 14:**
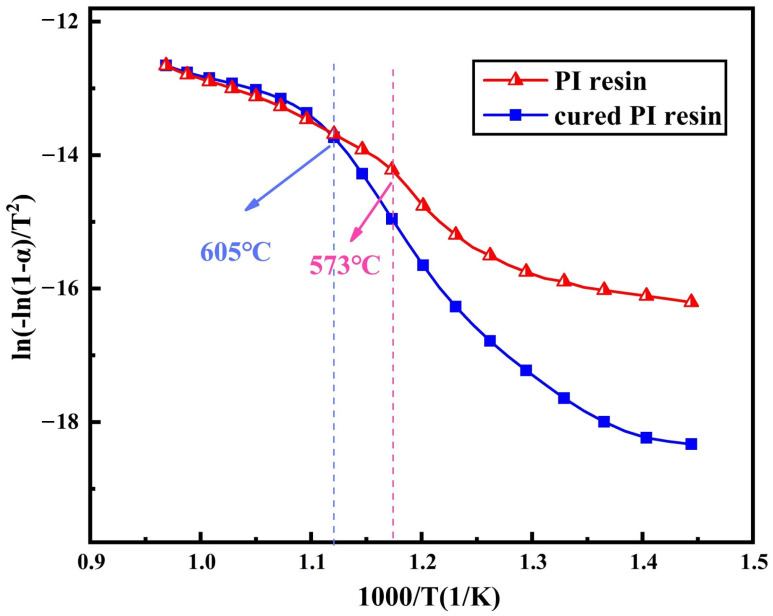
Thermal decomposition kinetics of PI resins and cured PI resins calculated by Coats–Redfern equation.

Figure 14 shows the Coats–Redfern relationship before and after the curing of PI resins treated according to the first-stage reaction. It can be seen that the Coats–Redfern diagram does not show a straight line relationship but has a turning point at the peak temperature. The results also show that the thermal decomposition process is completed in two steps. The thermal decomposition in the initial phase may be the thermal decomposition with the fatty chain in the copolymer, and the thermal decomposition in the later phase may be the thermal decomposition of the imide heterocyclic ring and the fatty ring generated by the reaction and the volatilization of the first thermal decomposition product. The activation energies of the two thermal decomposition reactions were calculated, respectively, and the results are listed in Table 3. The activation energy of the first thermal decomposition reaction is about 136~194 kJ/mol, which decreases obviously with the progress of the reaction, and in the second thermal decomposition reaction, the activation energy is 42~61 kJ/mol. Therefore, it can be inferred that the second thermal weightlessness process is mainly the high-temperature volatilization of the pyrolysis products in the first step. Compared with the first thermal decomposition reaction, the decomposition activation energy of PI after curing is obviously increased by 57 kJ/mol, while the second thermal decomposition activation energy is slightly lower than that before curing.

### 3.5. Molecular Dynamics Simulation of Thermal Decomposition Behavior

**Figure 15:**
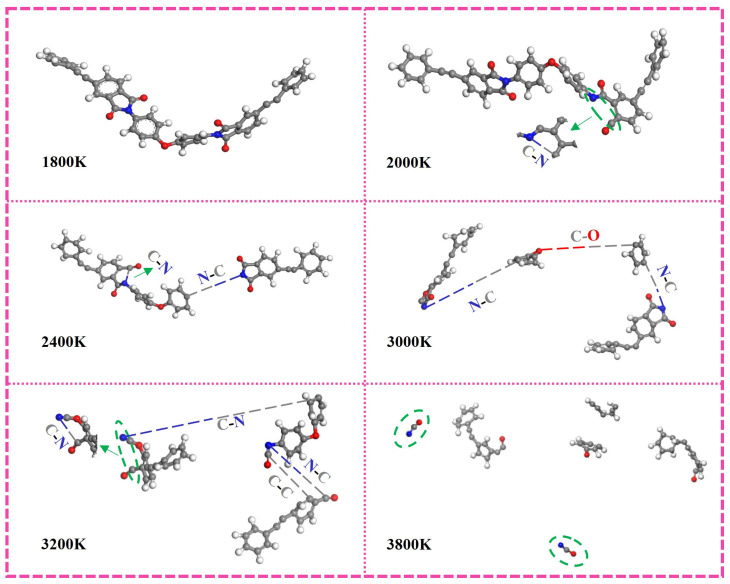
The initial reaction of the PI molecular chain at different temperatures (Among them, C atom-dark gray; H atom-light gray; O atom-red; N atom-blue).

Finding the initiation reactions of PI is important for analyzing its pyrolysis mechanism. The reaction force field of ReaxFF6.0 was used, and the reaction temperature was 2000 K to 3600 K, which was increased by 200 K in turn. The reaction process is shown in Figure 15. Below 2000 K, the molecular chain is only twisted, and there is no bond break. Above 3600 K, small molecular products began to appear after molecular chain decomposition. It can be seen that with the increase in temperature, all unstable single bonds are broken. The first one is C-N on the imide ring, followed by N-C connected to the benzene ring, and then C-O and C-C are also broken in turn.

**Figure 16:**
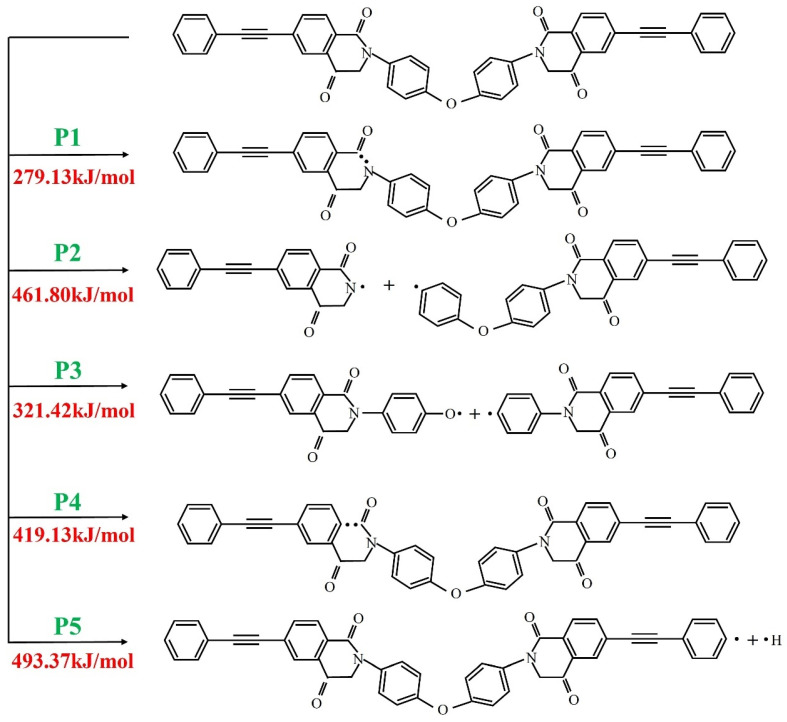
The main initiation reaction of PI resin pyrolysis.

According to the reaction initiated by PI, the bond energy of the fracture is calculated, and the result is shown in Figure 16. The energy of the C-N bond on the imide ring, which is also preferentially broken in the thermal decomposition reaction, is the lowest. Therefore, it is speculated that most of the thermal decomposition reactions begin with the breaking of this bond. However, the bonding energy of N-C linked to the benzene ring which breaks earlier in the reaction process is higher. It may be that in the actual reaction process, the benzene ring-containing product hinders the reaction, thus requiring a higher energy. With the distortion of the PI molecular chain under the influence of high temperature, the ether bond (C-O-C) with low bond energy began to break. Because of the distortion of the aromatic heterocyclic ring, the stability of the molecule is affected, and many C chains begin to form, which indicates that the main chain of the PI molecule capped by the phenylacetylene group begins to decompose. With the continuous progress of the reaction, benzene rings were destroyed one after another, and chemical bonds were continuously cracked, forming many macromolecular fragments with long molecular chains. Subsequently, these fragments continue to decompose until they are completely decomposed into small molecular products, and the thermal decomposition reaction is completed. In addition, the H radicals located outside the molecular chain structure will also have broken bonds, but some highly active H radicals will return to the main chain with the increase in temperature and the violent movement of the molecular chain to form C-H. However, what happens more in the process of the reaction is that H free radicals fall off at any time in the reaction.

## 4. Conclusions

Based on the curing process of phenylacetylene capped PI with excellent high-temperature resistance proposed by our research group, the curing mechanism and kinetics of PI were monitored by DSC technology, and its thermal decomposition mechanism and kinetics were analyzed by TGA technology. The DSC diagram shows that the top temperature of the PI resin is in the range of 370 °C~410 °C, which gradually increases with the increase in the heating rate. The apparent activation energy of the curing reaction calculated by the Kissinger method and Ozawa method is 157.5 kJ/mol and 160.2 kJ/mol, respectively. The reaction order calculated by the Crane method is 0.93. The kinetic parameters of the curing reaction were determined by the Friedman method, Starink method, and F-W-O method, respectively. The results show that the activation energy of the dynamic reaction can be divided into three stages, which is consistent with the molecular dynamics simulation of the changing stages of the curing reaction process. The TGA curves shows that when the mass loss of the cured products reached 5% and 20%, the corresponding thermal decomposition reaction temperature is 571 °C and 631 °C, and the decomposition peak temperature is 605 °C, and the *IPDT* is 2667.9 °C. The first thermal decomposition activation energy of PI resins and cured PI resins calculated by the Coats–Redfern method is 136.4 kJ/mol and 193.2 kJ/mol, and the second thermal decomposition activation energy is 60.7 kJ/mol and 42.3 kJ/mol, respectively. This shows that in the initial stage, there is the decomposition of the cyclic structure with high energy, and in the later stage of the reaction, there is a secondary decomposition of the initial decomposition product. In addition, the mechanism of initial thermal decomposition of PI resins was simulated by molecular dynamics, and it was found that the energy of the single bond was relatively low. The first one was C-N located on the imide ring, followed by N-C connected to the benzene ring, and then C-O and C-C also broke in turn. Among them, the bond-breaking energy of C-N located on the imide ring is the lowest, which is 279.13 kJ/mol. The bonding energy of the H radical located outside the molecular main chain is the highest, which is 493.37 kJ/mol.

## Figures and Tables

**Table 1:** Cure characteristic parameters of PI resins at different heating rates.

*β* °C/min	*T_i_* (°C)	*T_p_* (°C)	*T_f_* (°C)
5	346.8	372.7	388.1
10	359.5	391.1	405.1
15	362.7	398.0	420.2
20	369.5	403.1	423.8

Note: *T_i_* is the initial curing temperature, *T_p_* is the peak exothermic temperature and *T_f_* is the final curing temperature.

**Table 2:** TGA analysis of PI resins and cured PI resins.

State	*T*_5%_ (°C)	*T*_20%_ (°C)	*T_max_* (°C)	*RW* (%)	*IPDT*		
					A*	K*	*T* (°C)
PI resins	537	612	573	64.3	0.89	3.61	2127.7
cured PI resins	571	631	605	70.6	0.91	4.52	2667.9

A* and K* are determined by the area ratio of the total experimental curve defined by the total TGA thermal analysis diagram.

**Table 3:** Kinetic parameters of thermal decomposition obtained by Coats–Redfern method.

State	*E_a_*_1_	*R* ^2^	*E_a_*_2_	*R* ^2^
PI resins	136.4	0.988	60.7	0.982
Cured PI resins	193.2	0.998	42.3	0.976

## Data Availability

Supplemental data can be provided upon reasonable request.
